# Magnetic Resonance Imaging in Orbital Pathologies: A Pictorial Review

**DOI:** 10.5334/jbr-btr.1308

**Published:** 2018-01-04

**Authors:** Dilek Gokharman, Sonay Aydin

**Affiliations:** 1 Ankara Training and Research Hospital, TR

**Keywords:** MRI, orbita, diseases, mass

## Abstract

Orbital lesions form a wide range of pathologies, that create challenges in diagnosis, management, and treatment. The high-resolution soft tissue detail provided by magnetic resonance imaging (MRI) has allowed for better lesion characterization. Especially in cases where history and clinical evaluation are insufficient, MRI plays a crucial role. MRI is also important in the detection of the extent of orbital diseases. The aim of this study was to examine the MRI characteristics of common and/or rare diseases arising from or extending into the orbita to aid radiologists in the correct diagnosis of orbital lesions.

## Introduction

**Figure 1:**
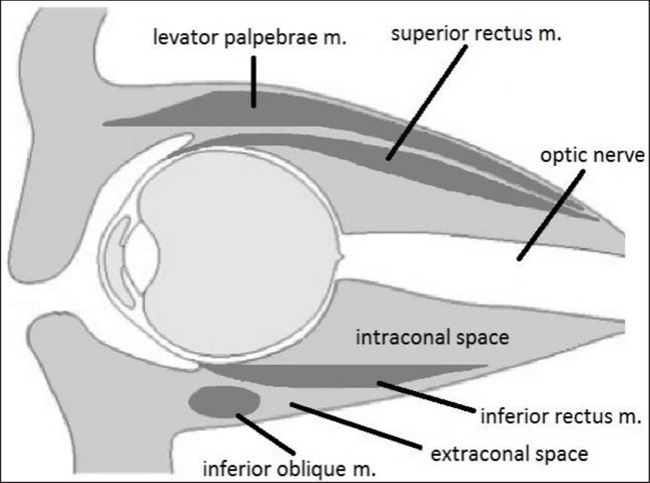
The orbital space compartments.

Orbital lesions form a wide range of pathologies that pose challenges in diagnosis, management and treatment. The soft tissue detail provided by magnetic resonance imaging (MRI) allows for better lesion characterization. MRI therefore plays a crucial role, especially in cases where history and clinical evaluation are inconclusive [1]. There are several approaches for the correct diagnosis of orbital pathology. A common diagnostic strategy is the localization of the pathology into the four main orbital compartments [2]: the ocular compartment or globe, the muscle cone and the intraconal and extraconal spaces (Figure 1). The muscle cone contains recti muscles and their fasciae; on its base, the globe is present, and the optic canal figures the apex. The globe is encircled by the Tenon’s capsule, which has three layers: the sclera, uvea and retina. The extraconal space includes the superior and inferior oblique muscles, levator muscle complex, the lacrimal gland and the orbital fat. The aim of this pictorial essay was to illustrate the MRI characteristics of common and/or rare diseases arising from or extending into the orbita, to help the radiologists to reach the correct diagnosis in orbital lesions.

This pictorial essay consists of cases of orbital pathologies collected from the Ankara Training and Research Hospital over a period of 10 years. MRI was performed with a GE 1.5T Signa and a Siemens 1.5T Magnetom-Aera scanner. The protocol included T1- and T2-weighted (T1-WI and T2-WI) oblique axial and sagittal images along the plane of the optic nerve, and true coronal T2-weighted images. When an intravenous contrast agent was administered, axial and coronal fat suppressed sequences were included.

## Ocular Pathologies

### Retinoblastoma

**Figure 2:**
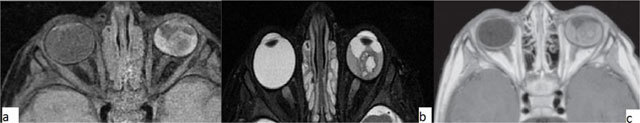
Retinoblastoma. A 4-year-old female with a left ocular lesion. The lesion is hyperintense on axial fat suppressed T1-WI **(a)** and hypointense on axial T2-WI. On contrast-enhanced T1-WI, mild contrast enhancement is seen.

Retinoblastoma originates from the retina in children younger than five-years of age, and may be hereditary or non-hereditary. Leukocoria is a common clinical finding (50%). Retinoblastoma is the most common primary intraocular tumour in children. Typical findings on non-contrast computed tomography (CT) is speckled calcification. MRI is useful for evaluating any possible extraocular or intracranial extension. Retinoblastoma appears hypointense on T2-WI and slightly hyperintense on T1-WI relative to the vitreous. After contrast, moderate enhancement is present. Heterogeneous enhancement and local thickening of the choroid adjacent to the tumour is a hallmark of choroidal invasion. MRI is the modality of choice for pre-treatment staging (Figure 2) [3,4].

### Choroidal metastases

**Figure 3:**
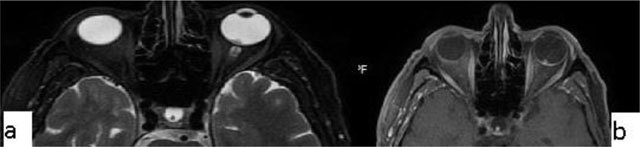
Choroidal metastasis (breast cancer metastasis). A 62-year-old female with a left ocular lesion. On axial fat suppressed T2-WI, choroidal thickening is seen near to the posterior margin of the globe **(a)**. After contrast injection, the lesion is well enhanced on axial, fat suppressed T1-WI **(b)**.

According to literature, the three most common sources of ocular metastases are the breast, lung and kidney cancer. The metastasis is generally located in the posterior segment. Carcinomas are the most common type of choroidal malignancies. It is important to keep in mind that dislocated lens may mimic choroidal metastasis of cutaneous malignant melanoma (Figure 3) [5,6,7].

### Choroidal melanoma

**Figure 4:**
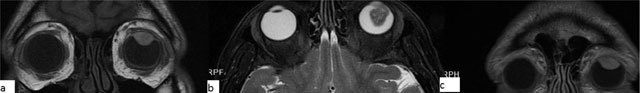
Choroidal melanoma. A 57-year-old male with a left ocular lesion. There is a 15 × 9 × 15 mm mass present on MRI of the orbita. The mass is hyperintense on T1-WI **(a)**, hypointense on T2-WI **(b)**, and shows significant contrast enhancement **(c)**. The histopathological analysis revealed a malignant melanoma.

Ocular melanoma is a malignant neoplasm that originates from the ciliary body, choroid or iris. The majority of lesions (90%) originate from the choroid. Ocular melanomas are the most common primary intra-ocular tumours in adults. They represent only 5% of all melanomas. Patients generally present in the 5th–6th decade of life (mean age, 56 years) [8]. Choroidal melanomas may be asymptomatic, or they can present with decreased vision, visual field defects, or floaters. The primary diagnostic method is fundoscopy. Imaging studies are generally used to determine the extent of the disease. MRI is superior to CT. Melanin has intrinsic T1 and T2 shortening effects, so that they present with increased T1-WI and decreased T2-WI signal intensity (Figure 3). MRI is also useful for identifying tumour size, extraocular extension, and ciliary body infiltration. In addition, MRI is better than CT in the identification of retinal detachment and extrascleral spread (Figure 4) [9].

### Coloboma

**Figure 5:**
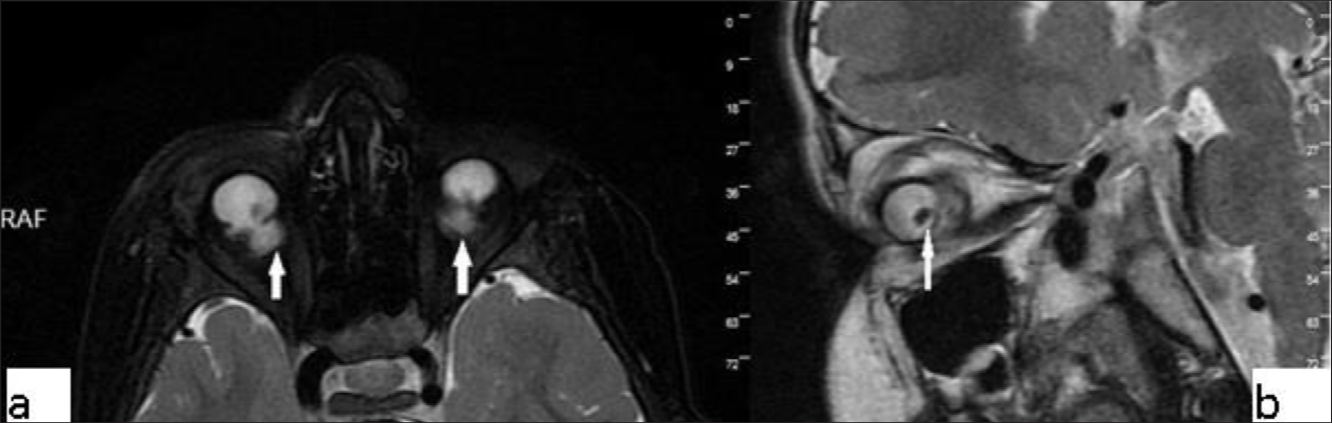
Coloboma. A 20-year-old male with congenital vision loss. On fat-suppressed axial T2-WI, the globes are small and irregular **(a)**. The posterior wall presents fluid intensity cysts (arrows). The dislocated lens is seen on sagittal T2-WI (**b**, arrow).

Coloboma is a congenital malformation that occurs due to the failure of fusion at the embryonic stage of the intraocular fissure, resulting in the absence of a part of the eye. On CT/MRI, a focal posterior defect in the globe with vitreous herniation is present. A retrobulbar fluid-density cyst may be seen (Figure 5) [10].

### Persistent hyperplastic primary vitrous (PHPV)

**Figure 6:**
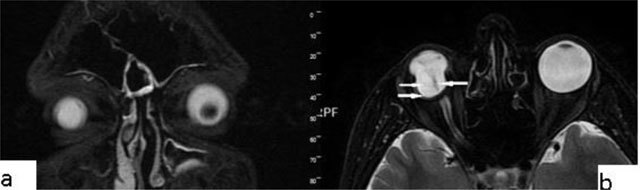
Persistent hyperplastic primary vitreous (PHPV). A 38-year-old male with a right ocular anomaly. On coronal fat-suppressed T2-WI there is right microphthalmia **(a)**. On coronal fat-suppressed T2-WI, the right orbita has a septum (**b**, arrow) and a colobomatous cyst (**b**, double arrow). The lens is absent.

PHPV refers to a rare congenital developmental malformation of the eye. It arises due to a failure of normal regression of the embryonic hyaloid vascular system. Commonly, PHPV manifests with leukocoria and microphthalmia in full-term infants. Generally on MRI the vitreous body is infiltrated with soft-tissue. In addition, after contrast administration, the vitreal lesions enhance (Figure 6) [11,12].

## Intraconal Lesions

### Optic nerve glioma

**Figure 7:**
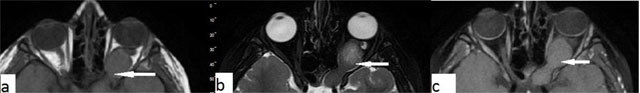
Optic nerve glioma. A 7-year-old male with a left intraconal lesion. The mass is isointense to the extraocular muscles) on axial T1-WI **(a)** and heterogenous-hyperintense on fat suppressed T2-WI **(b)**. Diffuse enhancement is seen **(c)**.

They are usually seen in the setting of neurofibromatosis type I (NF1). Almost all optic nerve gliomas are juvenile pilocytic astrocytomas and are seen in children < eight years of age. MRI is the method of choice. It is very useful in assessing involvement of the orbital apex, optic chiasm, hypothalamus, and other intracranial structures. The lesions are typically T1-WI isointense and T2-WI isointense to hyperintense. Enhancement is variable (Figure 7) [13,14].

### Optic neuritis

**Figure 8:**
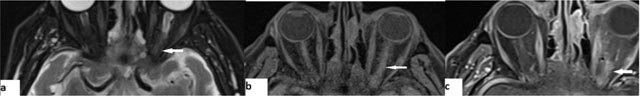
Optic neuritis. A 27-year-old-male with a left intraconal lesion. The optic nerve is seen slightly thick and hyperintens on axial T2-WI in its proximal intraconal portion (**a**, arrow). The T2-WI hyperintense area shows significant contrast enhancement on fat suppressed T1-WI (**c**, arrow).

Optic neuritis describes the inflammation of the optic nerve, including infectious and less frequently non-infectious causes. Characteristically, on imaging, optic neuritis is seen as unilateral optic nerve swelling in its retrobulbar/intra-orbital segment, with high T2-WI signal and contrast enhancement. Chronically, the optic nerve might become atrophied rather than swollen. In some cases, T2-WI hyperintensity might persist. Contrast enhancement is best detected with fat-suppressed T1-WI. Contrast enhancement is present in >90% of patients within 20 days of visual loss (Figure 8) [15].

### Meningioma

**Figure 9:**
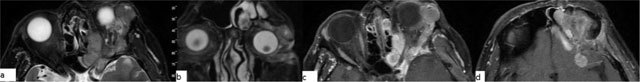
Meningioma. A 56-year-old male with a left intraconal lesion. On fat-suppressed images axial **(a)** and coronal **(b)** T2-WI, a heterogenous hyperintense mass is seen, filling the temporal fossa and orbita, extending into the frontal sinus. Post-contrast fat suppressed, axial T1-WI **(c, d)** show diffuse homogenous enhancement of the lesion.

Meningiomas can originate either from the optic nerve sheath or the periosteum of the orbital wall (primary meningioma), or secondarily, they can arise from the sphenoid ridge or tuberculum sellae and extend into the orbit. Meningiomas account for 2% of space-occupying orbital masses and they are the second most common optic nerve tumour. Secondary optic nerve meningiomas are more common than primary lesions. On both CT and MRI, fusiform enlargement of the optic nerve sheath is present. *Tram-track* enhancement along the sheath is an imaging characteristic for meningiomas (Figure 9). Differential diagnoses include sarcoidosis, Wegener’s granulomatosis and metastatic infiltration [8,16].

## Conal Lesions

### Thyroid ophthalmopathy

**Figure 10:**
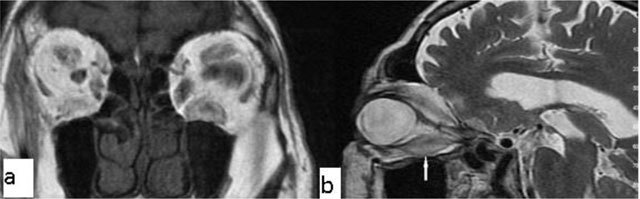
Thyroid ophthalmopathy. A 75-year old male with exophthalmos. On coronal T1-WI **(a)** the extraocular muscles are enlarged. Fusiform enlargement sparing the tendinous section, of the inferior rectus muscle is seen on sagittal T2-WI (**b**, arrow).

Enlargement of the extraocular muscles is the main presentation of thyroid ophthalmopathy (Figure 10). The inferior and medial recti are the most commonly involved. The tendinous portion of the affected muscle is typically spared (‘Coca-Cola bottle’ sign). At the initial phase of the disease the orbital fat is spared. Exophthalmos is the result of both muscle enlargement and hypertrophy of the retro-ocular fat [17].

### Orbital pseudotumor and orbital myositis

**Figure 11:**
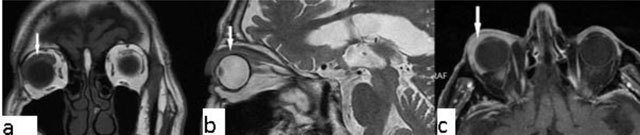
Orbital pseudotumor. A 45-year-old male with a right orbital lesion. The lesion is hypointense on both coronal T1 WI (**a**, arrow) and sagittal T2-WI (**b**, arrow). On contrast enhanced axial T1-WI, the lesion shows marked enhancement (**c**, arrow). It cannot be separated from the lacrimal gland that is enlarged, too.

Orbital pseudotumor is an acute inflammatory condition of the orbital soft tissues. It is one of the most common causes of unilateral exophthalmos. Pseudotumor can be distinguished from thyroid ophthalmopathy by involvement of the tendinous portion of the muscles, and the retro-orbital soft tissue. The process is generally hypointense. The lesion shows marked enhancement (Figure 11). Orbital lymphoma and metastasis are included in the differential diagnosis of orbital pseudotumor. Presenting with oblivious pain, obscure margins and rapid response to steroids are some of the features differentiating pseudotumor from real tumour lesions [18,19].

**Figure 12:**
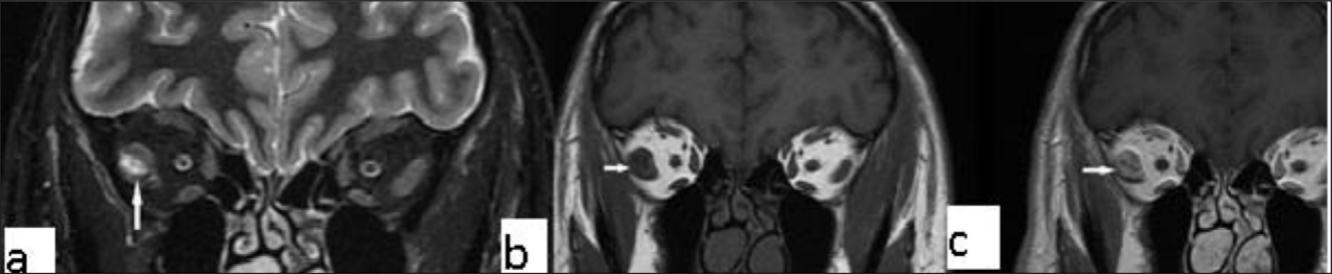
Orbital myositis. A 45-year-old female with a right orbital lesion. The lateral rectus is thickened and hyperintense on coronal fat supressed T2-WI (**a**, arrow). Coronal pre- and post-contrast T1-WI, showi increased enhancement of the lateral rectus (**b**, arrows).

Orbital myositis (Figure 12), is a non-infectious inflammatory condition. Myositis generally includes one or two extraocular muscles and present with thickening of the muscles involved. Contrast-enhanced, fat-suppressed T1-WI is the method of choice [20].

Immunoglobulin G4 (IgG4)-related disease is a systemic inflammatory process of unknown etiology, characterized by tissue infiltration by IgG4 plasma cells. In patients with extraocular muscle enlargement, sparing tendons IgG4–related disease should be kept in mind as a leading differential diagnosis, especially when the lateral rectus is the most enlarged muscle [21].

## Extraconal Lesions

### Dacryocystocele

**Figure 13:**
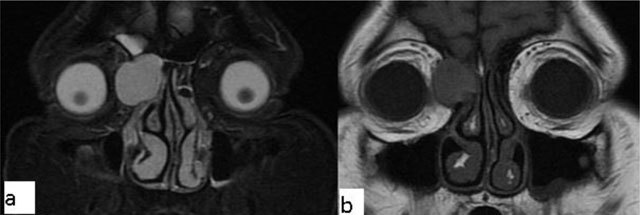
Dacryocystocele. A 50-year-old female with a left orbital lesion. The lesion is hyperintense on fat suppressed coronal T2-WI **(a)** and isointense, because of concurrent infectious process, on fat suppressed coronal T1-WI **(b)**.

Dacryocystocele occurs when tears accumulate in the lacrimal sac or lacrimal canal proximal to an obstruction in the lacrimal canal. The classic MRI appearance is similar to that of simple cysts if the content is homogenous, hyperintense on T2-WI and hypointense on T1-WI. Complicated (e.g. infectious) cysts may exhibit more heterogenous content, though there is generally no enhancement (Figure 13) [22].

### Tolosa-Hunt syndrome

**Figure 14:**
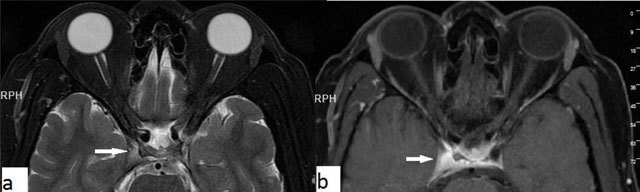
Tolosa-Hunt syndrome. A 50-year-old male with right cavernous sinus lesion (arrows). It appears hyperintense on T2-WI **(a)** and after contrast injection on fat saturated T1-WI **(b)**.

Tolosa-Hunt syndrome is a recurrent, idiopathic, painful inflammatory condition caused by inflammation of the cavernous sinus or superior orbital fissure. On MRI, the lesion can be iso to hypointense on T1-WI and hypo/hyperintense on T2-WI (Figure 14). After contrast administration, expansion of the cavernous sinus is seen, with enhancement of the soft tissue mass. The condition is often successfully amenable to steroid treatment [23].

### Dermoid and epidermoid cysts

**Figure 15:**
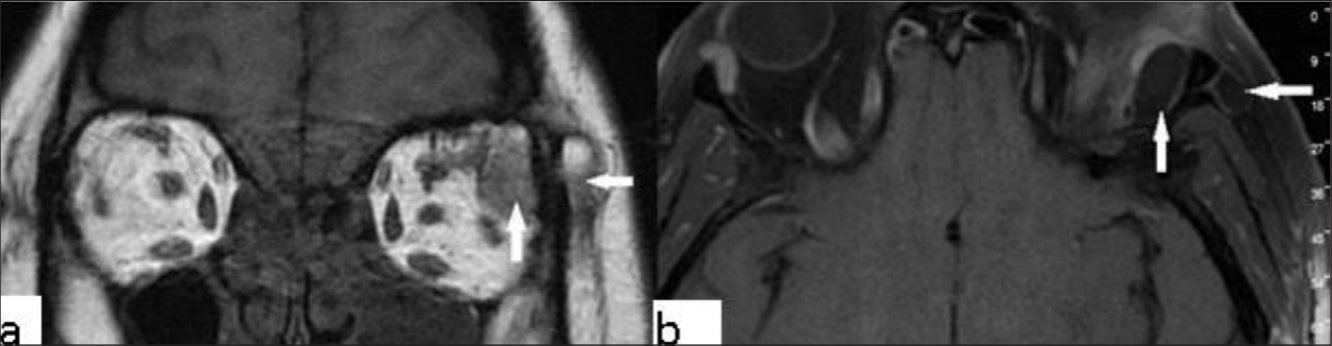
Dermoid cyst. A 33-year-old female with a left orbital lesion. On coronal T1-WI, the lesion is heterogeneous and hyperintense (**a**, arrows). On fat suppressed axial post-contrast T1-WI, complete signal suppression is present, indicating fatty content without contrast enhancement (**b**, arrows).

**Figure 16:**
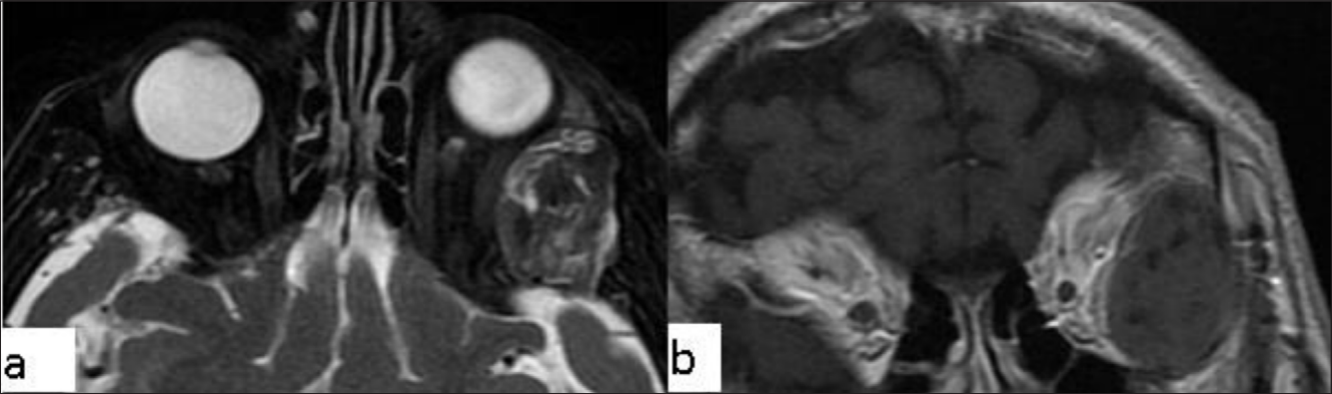
Epidermoid cyst. A 75-year-old male with a left orbital lesion. On sagittal fat suppressed T2-WI the lesion is heterogeneous and hyperintense **(a)**. After contrast administration the lesion does not show enhancement on coronal T1-WI **(b)**.

Intraorbital dermoid cysts represent 5–10% of all dermoid cysts, while intraorbital epidermoids are more rarely observed. Histologically, both are lined by squamous epithelium. Differently, dermoid cysts include all three germ layers and are characterized by the presence of mesodermal elements such as hair follicles. Dermoid cysts usually show diffusion restriction on diffusion-weighted images. They do not enhance after contrast injection, have smooth margins, and cystic/solid components. Sometimes calcifications may be suspected (Figures 15 and 16) [24,25].

## Vascular Lesions

### Cavernous hemangioma

**Figure 17:**
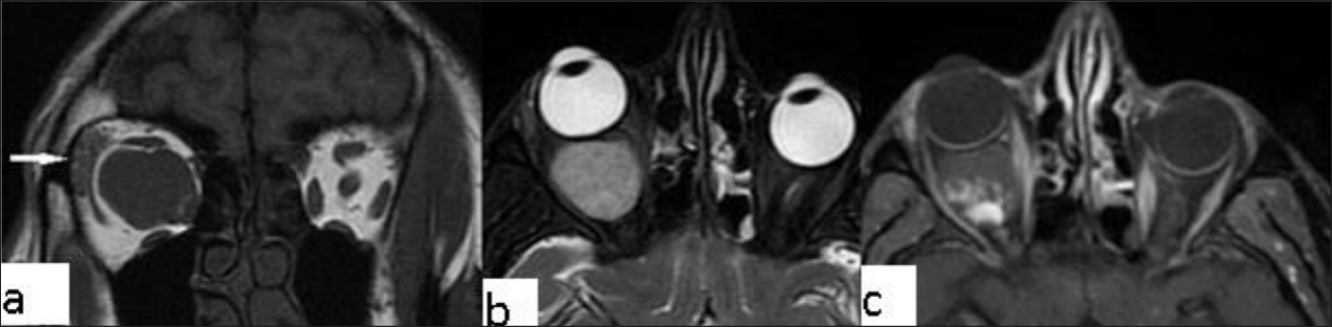
Cavernous hemangioma. A 43-year-old female with a right orbital lesion. The lesion is isointense to the muscles on coronal T1-WI (**a**, arrow), and hyperintense on fat suppressed coronal T2-WI **(b)**. After contrast administration, avid enhancement is seen on coronal T1-WI **(c)**.

Cavernous haemangioma is the most common benign intraorbital lesion in adults. It generally presents in the second to fifth decades. Painless, slowly progressive, proptosis is the most common complaint. They generally locate to the intraconal space. They are homogenous masses with smooth margins, uniform enhancement is common. They can be easily separated from the optic nevre and extraocular muscles. The orbital apex is usually spared (Figure 17).

Capillary hemangiomas are the main part of the differential diagnosis. They are the most common orbital tumours of infancy. The MRI characteristics are slight hypointensity on T1, iso- to hyperintensity on T2 with multiple serpiginous flow voids. Homogenous enhancement is common. Lobulated appearance with thin septa is characteristic for capillary hemangiomas [26].

## Conclusion

A compartmental approach to evaluating orbital disease can guide the differential diagnosis. Use of MRI is valuable for determining the extent of disease, describing its exact localisation and detecting involved orbital compartments. MRI is also a useful diagnostic tool for both orbital and intracranial pathologies. Indeed, intracranial diseases can present with orbital symptoms or as orbital masses, likewise orbital pathologies can extend into the cranium and present with central nervous system symptoms. In addition, with the guidance of MRI, possible involvement of the orbital apex and associated intracranial abnormalities can be shown.
